# P-1968. Mask fatigue, compliance, and illness among healthcare workers during the COVID-19 pandemic

**DOI:** 10.1093/ofid/ofae631.2127

**Published:** 2025-01-29

**Authors:** Ria Patel, Aparna Jayaram, Mirza Ali, Alfredo J Mena Lora

**Affiliations:** Ross University, Rockville, Maryland; Ross University, Rockville, Maryland; Saint Anthony Hospital, Chicago, Illinois; University of Illinois Chicago, Chicago, Illinois

## Abstract

**Background:**

During the COVID-19 pandemic, healthcare facilities frequently adopted mandatory masking to protect patients and healthcare workers (HCWs). Although effective in preventing virus spread, prolonged masking can induce discomfort and fatigue among HCWs. As the pandemic progressed, the prolonged use of masks became a source of growing discomfort and presented increasing challenges with masking policy compliance. There is a paucity of data on masking fatigue among HCWs.

**Figure:**
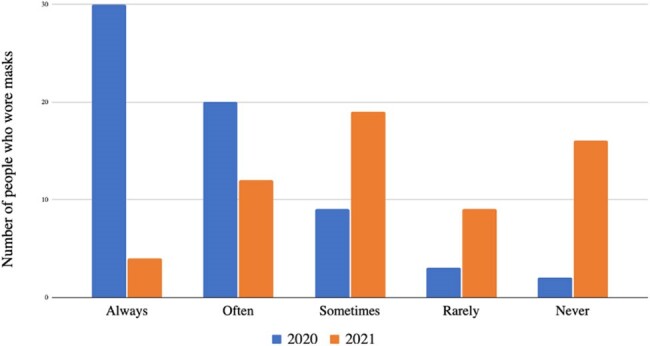

Mask use in HCWs with mask fatigue

**Methods:**

We performed an anonymous survey of at two hospitals in Chicago. An anomyous survey was sent via hospital list-servs to all HCWs. The survey was sent on May 23, 2023 and collected the clinical roles of participants, masking habits during the pandemic, masking fatigue, and self-reported influenza-like illness (ILI). Data was tabulated and descriptive statistics used.

**Figure:**
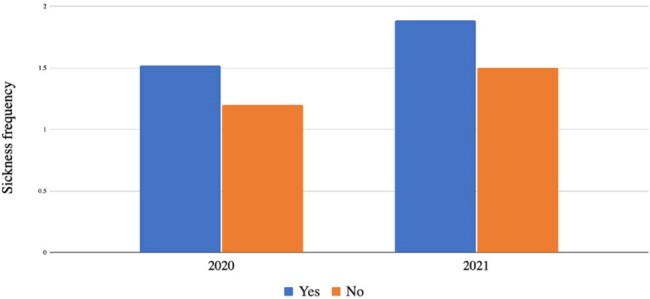

Differences in sickness frequency as a correlate of mask fatigue

**Results:**

A total of 90 HCWs provided responses, with 66% experiencing mask fatigue throughout the study period. In 2020, among HCWs who reported mask fatigue, 46% of always wore their masks, while 3% never wore masks (Figure 1). In 2021, HCWs who always wore their masks decreased to 6%, while the percentage of HCWs who never wore their masks increased to 26% (p=0.0075). An statistically significant increase (p=0.0312) in reported ILI was seen among HCWs with mask fatigue in 2021 (Figure 2).

**Conclusion:**

Masking fatigue was common during the pandemic and increased with time. Reduced masking due to this fatigue may have led to increased ILI in HCWs. Fatigue and non-compliance can contribute to HCW absenteeism and transmission to vulnerable patients. Finding strategies to mitigate mask fatigue and ensure consistent adherence to masking policies is critical during respiratory virus season or future respiratory pandemics.

**Disclosures:**

All Authors: No reported disclosures

